# Pregnancy glucagon-like peptide 1 predicts insulin but not glucose concentrations

**DOI:** 10.1007/s00592-023-02142-8

**Published:** 2023-07-13

**Authors:** Danielle L. Jones, Clive J. Petry, Keith Burling, Peter Barker, Elizabeth H. Turner, Laura C. Kusinski, Claire L. Meek

**Affiliations:** 1 Metabolic Research Laboratories, Wellcome-MRC Institute of Metabolic Science, University of Cambridge, Cambridge, CB2 0QQ UK; 2 NIHR Biomedical Research Centre Core Biochemistry Assay Lab, University of Cambridge, Addenbrooke’s Hospital, Cambridge, CB2 0QQ UK; 3 Department of Clinical Biochemistry, Addenbrooke’s Hospital, Cambridge, UK; 4 Wolfson Diabetes and Endocrinology Clinic, Cambridge University Hospitals, Addenbrooke’s Hospital, Cambridge, UK

**Keywords:** Incretin, Insulin secretion, Insulin sensitivity, Gestational diabetes, Obesity

## Abstract

**Aims:**

Incretin hormones glucagon-like peptide 1 (GLP-1) and gastric inhibitory peptide (GIP) cause increased insulin secretion in non-pregnant adults, but their role in pregnancy, where there are additional metabolically-active hormones from the placenta, is less clear. The aim of the present study was to assess if fasting and post-load incretin concentrations were predictive of pregnancy insulin and glucose concentrations.

**Methods:**

Pregnant women (n = 394) with one or more risk factors for gestational diabetes were recruited at 28 weeks for a 75 g oral glucose tolerance test (OGTT). Glucose, insulin, GLP-1 and GIP were measured in the fasting state and 120 min after glucose ingestion.

**Results:**

Fasting plasma GLP-1 concentrations were associated with plasma insulin (standardised β’ 0.393 (0.289–0.498), *p* = 1.3 × 10^–12^; n = 306), but not with glucose concentrations (*p* = 0.3). The association with insulin was still evident when adjusting for BMI (β’ 0.271 (0.180–0.362), *p* = 1.1 × 10^–8^; n = 297). Likewise, at 120 min the OGTT GLP-1 concentrations were associated with plasma insulin concentrations (β’ 0.216 (0.100–0.331), *p*  = 2.7 × 10^–4^; n = 306) even after adjusting for BMI (β’ 0.178 (0.061–0.294), *p* = 2.9 × 10^–3^; n = 296), but not with glucose (*p*  = 0.9). GIP concentrations were not associated with insulin or glucose concentrations at either time point (all *p*  > 0.2). In pregnancy plasma GLP-1, but not GIP, concentrations appear to be predictive of circulating insulin concentrations, independently of associations with BMIs.

**Conclusions:**

These results suggest that the relationship between insulin and incretins is preserved in pregnancy, but that other factors, such as placental hormones or counter-regulatory hormones, may be more important determinants of glycaemia and gestational diabetes aetiology.

## Introduction

The incretins glucagon-like peptide 1 (GLP-1) and gastric inhibitory peptide (GIP) are secreted from L- and K-cells of the gastrointestinal tract, respectively, in response to the ingestion of glucose and certain other nutrients such as amino acids and fatty acids. Increased circulating concentrations leads to the stimulation of pancreatic β-cells to secrete insulin [1]. They signal through specific receptors, the GLP-1 receptor (GLP-1R) and the GIP receptor (GIPR), respectively. In addition to pancreatic β-cells, these receptors are expressed in tissues such as adipose tissue, bone, kidney, heart, lungs, gastrointestinal tract, immune system and specific areas of the brain [2, 3]. This widespread expression explains some of the broad functions of GLP-1 and GIP which are additional to the stimulation of insulin secretion, such as increased bone formation (and decreased absorption), stimulation of memory, inhibition of appetite (at pharmacological doses), increases in fat accumulation, increases in cardioprotection and cardiac output, decreases in gastric acid secretion and gastric emptying, and decreases in renal sodium excretion [2, 4].

Whilst these functions were established outside of pregnancy, the importance of the incretins in pregnancy [5, 6], where there are additional hormones secreted by the placenta, has yet to be fully established. The placenta does not express the incretin receptors GLP-1R and GIPR [7], but dipeptidyl peptidase IV, an enzyme that cleaves GLP-1 and GIP to inactive forms and therefore is key to their regulation, is highly expressed there [8]. Given that dipeptidyl peptidase IV is thought to be largely responsible for the very short half-life of circulating GLP-1 and GIP (2–3 and 4–5 min, respectively [2]) it is possible that the additional dipeptidyl peptidase IV activity provided by the placenta during pregnancy may change the functional importance of incretins.

Studies of incretins in pregnancy have largely been focussed on circulating GLP-1 and GIP concentrations in gestational diabetes, hyperglycaemia that first develops or is first recognised during pregnancy. Gestational diabetes is believed to result from inadequate insulin secretion failing to compensate for the degree of pregnancy insulin resistance. Results from these studies have been somewhat inconsistent: gestational diabetes has been associated with increased fasting GLP-1 concentrations but not post-load GLP-1 concentrations or GIP concentrations at any time point in the oral glucose tolerance test (OGTT) [9], increased post-load but not fasting GLP-1 concentrations [10], lower OGTT 180 min GLP-1 concentrations but no difference in fasting concentrations [11], reduced fasting GLP-1 and GIP concentrations [12], reduced fasting GLP-1 but not GIP concentrations, and unchanged GLP-1 and GIP areas under the OGTT curve [13], reduced OGTT 30 min but unchanged fasting GLP-1 concentrations [14], reduced incretin effects [15] and unchanged fasting incretin concentrations [16]. Although the results of studies investigating the roles of GLP-1 and GIP in pregnancy therefore appear quite variable, the strength of effects on insulin secretion are so strong outside of pregnancy that it has been suggested that drugs that prolong the half-life of circulating incretins may be suitable treatments for gestational diabetes [17].

The present study was performed to investigate if fasting and post-load OGTT GLP-1 and GIP concentrations were predictive of pregnancy insulin and glucose concentrations at the beginning of the third trimester of pregnancy.

## Methods

### Participants, testing & diagnosis

The ongoing, multicentre OPHELIA Study (Observational study of Pregnancy Hyperglycaemia, Endocrine causes, Lipids, Insulin and Autoimmunity) is a prospective observational study that started recruiting from October 2019 [18]. Women were recruited to the study if they had a singleton pregnancy and one or more risk factors for gestational diabetes, as defined by the UK National Institute of Clinical Excellence (NICE) guideline NG3 [19]. The participants underwent a standard 75 g OGTT which took place between weeks 24 and 28 of pregnancy. Blood samples were collected after an overnight fast, and 120 min after the oral consumption of 75 g glucose. Samples for insulin and incretins were placed on ice, centrifuged and then separated from red blood cells within 30 min of venepuncture. They were then frozen and stored at − 80 °C for later batch analysis. Gestational diabetes was diagnosed using the OGTT glucose concentrations and NICE criteria (fasting sample ≥  5.6 mmol/L and/or 120 min sample  ≥  7.8 mmol/L) [19].

The present analysis was performed using samples and data from women recruited to the OPHELIA Study. These women were recruited from seven different centres in the UK (77 from Hinchingbrooke Hospital, Cambridgeshire; 13 from Queen Charlotte’s and Chelsea Hospital, London; 88 from Lister Hospital, Stevenage, Hertfordshire; 46 from Colchester General Hospital, Essex; 45 from Norfolk & Norwich University Hospital, Norfolk; 106 from Peterborough City Hospital, Peterborough; 8 from Ipswich Hospital, Suffolk).

### Ethical approval

Ethical approval for the OPHELIA Study was granted by the London – Westminster Research Ethics Committee, London, U.K. (18/LO/0477). All procedures followed were in accordance with both institutional and international guidelines. Written informed consent was obtained from all participants.

### Assays

Glycated haemoglobin (HbA1c) and glucose concentrations were measured locally in accredited laboratories, predominantly using Tosoh high performance liquid chromatography and glucose oxidase assays, respectively. GLP-1 (total) was measured in EDTA plasma using Meso Scale Discovery (Meso Scale Diagnostics, Rockville, Maryland, U.S.A.) immunoassay kits [20]. Intra-assay CVs were 5.2–8.2% and the working range of the assay was 1.4–1000 pg/mL. GIP (total) concentrations were measured in a subset of the samples where GLP-1 was measured, according to sample availability. They were also measured using Meso Scale Discovery immunoassay kits [20]. Intra-assay CVs were 9.3–11.0% and the working range of the assay was 1.0–2500 pg/mL. Insulin concentrations were measured in heparinised plasma using Diasorin Liaison chemiluminescent immunoassay kits (Diasorin LTD., Dartford, Kent, U.K.). Intra-assay CVs were 5.0–6.0% and the working range of the assay was 3–3000 pmol/L.

### Calculations

The body mass index (BMI) was calculated as the mother’s weight (either pre-pregnancy or at the OGTT) divided by the height squared. The Matsuda index (an index of insulin sensitivity [21, 22]) and the Stumvoll index (an alternative index of insulin sensitivity [23, 24]) were calculated. The area under the OGTT curve and insulin to glucose ratio (an index of beta cell function and insulin secretion [25]) were calculated.

### Statistical analysis

Associations between two continuous variables were tested by linear regression, both unadjusted and adjusted for potential confounders (e.g. BMI). If necessary, hormone concentrations were natural log-transformed prior to analysis to ensure that the statistical model residuals were normally distributed. Standardised normal probability plots were used to assess if the residuals were normally distributed. In general, our analysis used glucose and insulin as linear variables, to remove the variability posed by the differing diagnostic criteria for gestational diabetes in clinical use internationally. However, where associations with binary variables such as gestational diabetes were required, we tested these using logistic regression. Chi-squared or Fisher’s exact tests were used to test associations between categorical variables. Missing data were managed by pairwise deletion. *P* < 0.05 was considered statistically significant throughout. Statistical analyses were performed using Stata version 16.1 (Stata Corp., from Timberlake Consultants Ltd., Richmond, Surrey, UK). Data are mean (SD) unless stated otherwise. We chose to present our data graphically as quintiles way to show the linear relationships between variables, since mean values for quintiles more closely followed the continuous distribution of GLP-1 concentrations.

## Results

### Clinical characteristics

Clinical characteristics of the participants are shown in Table 1. We recruited a total of 394 women. Ethnically, 84.8% (n = 334) of the participants classified themselves as White, 3.8% (n = 15) as Black, and 9.9% (n = 39) as Asian. The remaining 1.5% (n = 6) classified themselves as belonging to other ethnic groups. Most women were obese with a pre-pregnancy BMI of  > 30 kg/m^2^ (n = 198; 51.5%), aged over 30 years old (n = 231; 58.7%) and had at least one previous pregnancy (n = 242; 61.4%). The majority of women had normal glucose tolerance, according to fasting (participant mean (SD) 4.4 (0.4) mmol/L) and 120 min (5.8 (1.3) mmol/L) glucose concentrations during the OGTT.

**Table 1:** Clinical characteristics and analyte concentrations of the participants from this analysis

Clinical characteristic	n	Mean (SD) (range)
Maternal age (years)	371	31.3 (4.9)
		(19.1–45.1)
Parity (n nulliparous (%))	394	152 (38.6%)
Pre-pregnancy BMI (kg/m^2^)	392	30.3 (7.2)
		(14.3–56.3)
BMI at OGTT (kg/m^2^)	382	33.0 (7.0)
		(18–58)
Gestational diabetes (n (%))	394	34 (8.6%)
HbA1c (mmol/mol)	387	32.1 (3.4)
		(20–49)
OGTT fasting glucose concentration (mmol/L)	394	4.4 (0.4)
		(3.4–6.4)
OGTT 120 min glucose concentration (mmol/L)	390	5.8 (1.3)
		(2.6–11.5)
OGTT fasting insulin concentration (pmol/L)	302	86.2 (51.1)
		(7–388)
OGTT 120 min insulin concentration (pmol/L)	303	493.6 (358.3)
		(17–2003)
OGTT fasting GLP-1 concentration (pg/mL)	394	19.6 (11.2)
		(6–167)
OGTT 120 min GLP-1 concentration (pg/mL)	378	18.2 (11.2)
		(4–145)
OGTT fasting GIP concentration (pg/mL)	62	65.2 (38.2)
		(17–208)
OGTT 120 min GIP concentration (pg/mL)	61	303.3 (137.5)
		(73–929)

Continuous variable data are mean (SD) (range)

### Associations between OGTT incretin concentrations, glucose and insulin

The fasting and OGTT 120 min GLP-1 concentrations significantly correlated with each other (r = 0.40, *p* < 0.0001, n = 384). Similarly, the fasting and OGTT 120 min GIP concentrations were also significantly correlated (r = 0.48, *p* = 0.0001, n = 61). Fasting GLP-1 concentrations did not significantly correlate with fasting GIP concentrations (r = 0.22, *p* = 0.08, n = 62). Neither did the equivalent OGTT 120 min concentrations correlate significantly (r = 0.19, *p* = 0.1, n = 62).

**Fig. 1:**
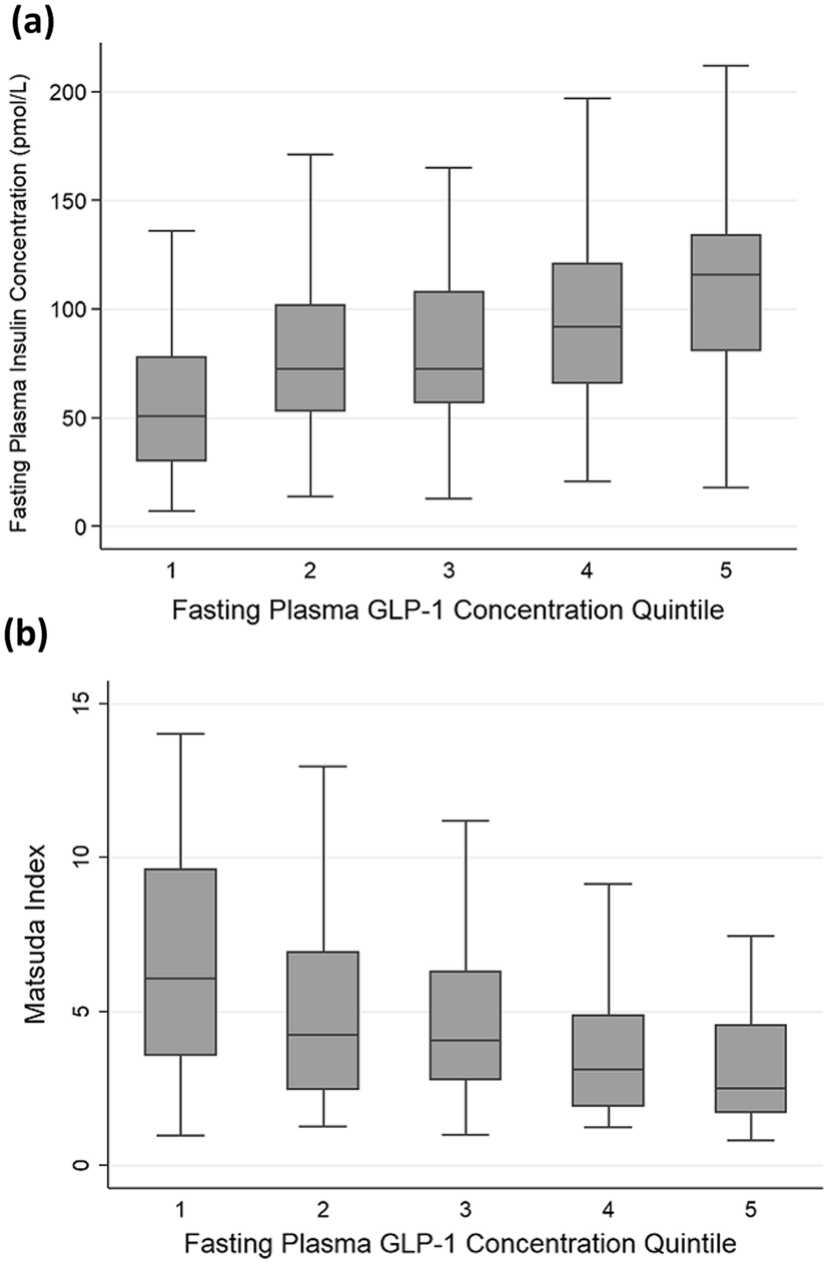
Box plots of quintiles of fasting plasma GLP-1 concentrations and **a** fasting plasma insulin concentrations and **b** Matsuda indexes, showing the median, interquartile range and range (excluding outliers defined as less than the lower quartile minus 3 times the interquartile range or more than the upper quartile plus 3 times the interquartile range) in each case

Fasting plasma GLP-1 concentrations were associated with fasting insulin concentrations and all the indices of insulin secretion and sensitivity that were tested (Table 2). Quintiles of GLP-1 concentrations were positively associated with fasting plasma insulin concentrations (*p* = 3.1 × 10^–12^; n = 306) and negatively with Matsuda indexes (*p* = 1.6 × 10^–10^; n = 299) (Fig. 1). They were also negatively associated with Stumvoll indexes (*p* = 6.4 × 10^–9^; n = 290). Fasting plasma GLP-1 concentrations were not associated with fasting glucose concentrations (Table 2). OGTT 120 min GLP-1 concentrations were associated with post-load plasma insulin concentrations and the related indexes but not with concurrent glucose concentrations (Table 2). Plasma GIP concentrations in the fasting or post-load state were not associated with either fasting insulin or glucose concentrations, or with the indexes of insulin secretion or sensitivity (Table 2).

**Table 2:** Associations between OGTT plasma incretin hormone concentrations and glucose and insulin concentrations and indexes of insulin secretion and sensitivity

	n	Standardised β	*p*-value
*(a) GLP-1 0 min*			
Glucose 0 min	394	0.047 (− 0.051–0.145)	0.3
Insulin 0 min	306	0.393 (0.289–0.498)	1.3 × 10^–12^
AUC Insulin/AUC Glucose ratio	263	0.243 (0.122–0.364)	9.9 × 10^–5^
Matsuda index	299	− 0.341 (− 0.449– − 0.232)	2.2 × 10^–9^
Stumvoll index	290	− 0.323 ( − 0.435– − 0.211)	3.1 × 10^–8^
*(b) GLP-1 120 min*			
Glucose 120 min	385	0.002 (− 0.097–0.100)	0.9
Insulin 120 min	306	0.216 (0.100–0.331)	2.7 × 10^–4^
AUC Insulin/AUC Glucose ratio	262	0.270 (0.148–0.392)	1.9 × 10^–5^
Matsuda index	298	− 0.238 (− 0.356– − 0.121)	7.7 × 10^–5^
Stumvoll index	289	− 0.183 (− 0.305– − 0.060)	3.7 × 10^–3^
*(c) GIP 0 min*			
Glucose 0 min	62	− 0.082 (− 0.418–0.255)	0.6
Insulin 0 min	41	0.197 (−0.157–0.551)	0.3
AUC Insulin/AUC Glucose ratio	38	0.058 (−0.179–0.296)	0.6
Matsuda index	41	− 0.102 (− 0.392–0.188)	0.5
Stumvoll index	37	− 0.091 (− 0.387–0.204)	0.5
*(d) GIP 120 min*			
Glucose 120 min	62	0.089 (− 0.247–0.426)	0.6
Insulin 120 min	41	0.093 (− 0.155–0.341)	0.5
AUC Insulin/AUC Glucose ratio	38	0.070 (− 0.195–0.336)	0.6
Matsuda index	41	0.184 (− 0.113–0.481)	0.2
Stumvoll index	37	− 0.071 (− 0.412–0.271)	0.7

Standardised β values are presented as mean (95% confidence interval)

Gestational diabetes was diagnosed in 34 (8.5%) of the participants. GLP-1 concentrations were not associated with gestational diabetes diagnosis in the fasting (odds ratio 0.97 (0.92‒1.02), *p* = 0.2, n = 394) or the post-load (odds ratio 0.99 (0.95‒1.03), *p* = 0.6, n = 390) states. Similarly, gestational diabetes was not associated with GIP concentrations, either fasting (odds ratio 1.01 (0.99‒1.02), *p* = 0.5, n = 62 (29 of whom had gestational diabetes)) or at 120 min after the glucose load (odds ratio 1.00 (1.00‒1.00), *p* = 0.8, n = 62 (30 of whom had gestational diabetes)).

### Associations between maternal BMI and OGTT incretin concentrations, and its influence on insulin-GLP1 associations

Pre-pregnancy BMI was not associated with gestational diabetes (odds ratio 0.99 (0.94–1.04); *p* = 0.7; n = 394) and neither was obesity (defined as pre-pregnancy BMI > 30 kg/m^2^; odds ratio 0.94 (0.46–1.89); *p* = 0.9; n = 394). The BMI at OGTT was positively associated with plasma GLP-1 concentrations in the fasting state (standardised β = 0.15 (0.05‒0.25), *p* = 2.8 × 10^–3^, n = 383) but not post-load (standardised β = 0.07 ( − 0.03‒0.18), *p* = 0.2, n = 376). BMI at OGTT was not associated with fasting GIP concentrations (standardised β =  − 0.02 (− 0.27‒0.23), *p* = 0.9, n = 61). It was, however, negatively associated with 120 min GIP concentrations (standardised β =  − 0.32 ( − 0.55‒ − 0.08), *p* = 9.9 × 10^–3^, n = 61).

When adjusting the associations between fasting GLP-1 concentrations and insulin concentrations and indexes for BMI, there were still significant associations with fasting insulin concentrations (standardised β = 0.277 (0.185‒0.369), *p* = 9.2 × 10^–9^, n = 297), the ratio of OGTT insulin to glucose areas under the curve (standardised β = 0.215 (0.095‒0.335), *p* = 5.1 × 10^–4^, n = 259), the Matsuda index (standardised β =  − 0.254 ( − 0.358‒ − 0.149), *p* = 2.7 × 10^–6^, n = 294) and the Stumvoll index (standardised β = -0.250 (-0.360‒ -0.141), *p* = 1.0 × 10^–5^, n = 285). Similarly, after adjusting for the BMI, the OGTT 120 min GLP-1 concentrations were still significantly associated with the 120 min insulin concentrations (standardised β = 0.178 (0.061‒ 0.295), *p* = 3.0 × 10^–3^, n = 296), the ratio of OGTT insulin to glucose areas under the curve (standardised β = 0.222 (0.100‒0.343), *p* = 3.8 × 10^–4^, n = 258), the Matsuda index (standardised β =  − 0.175 ( − 0.285‒ − 0.028), *p* = 0.02, n = 293) and the Stumvoll index (standardised β =  − 0.146 ( − 0.263‒ − 0.065), *p* = 1.9 × 10^–3^, n = 284).

## Discussion

Plasma GLP-1 concentrations in the third trimester of pregnancy are strongly predictive of insulin concentrations (and indices of insulin secretion and resistance) but not of glucose concentrations or the presence of gestational diabetes. Taken together, these results suggest that the relationship between insulin and incretins is preserved in pregnancy, but that other factors are more important as determinants of glycaemia.

### Strengths and limitations of the study

The strengths of this study include the large number of participants having circulating GLP-1 concentrations measured. Other strengths of the study are its multicentre recruitment leading to potentially enhanced reproducibility and generalisability, and the very careful sample handling to optimise the accurate measurement of GLP-1 and GIP concentrations. The limitations of this study include the fact that circulating GIP concentrations were measured in a much smaller number of samples than GLP-1 (due to financial constraints), lowering the statistical power to find significant associations. The OGTT time points when concentrations of GLP-1 and GIP were measured may also not have been optimal for assessing incretin function, peak concentrations having been reported to be around 15–30 min post-load in a non-pregnant population [18]. These factors may have contributed to the general lack of significant associations with GIP concentrations. However, the number of participants in which GIP concentrations were measured were at least comparable to those in a number of other studies of incretin concentrations in gestational diabetes [9, 12, 13]. Another limitation of the present study was the relatively low proportion of participants with gestational diabetes, which could have limited the statistical power to find significant associations with this condition. In this study, 8.6% of women were diagnosed with gestational diabetes, perhaps slightly lower than the expected incidence of around 10% in a high-risk population. We note that there is no current national data on the incidence of gestational diabetes. However, given that the main associations found in this study relate to pregnancy rather than gestational diabetes *per se*, the key conclusions of this study were unaffected by this limitation. Finally, insulin sensitivity and secretion were assessed using surrogate indices in this study, rather than gold standard methods such as euglycaemic and hyperglycaemic hyperinsulinaemic clamps. We consider that the ‘gold standard’ methods such a euglycaemic and hyperglycaemic hyperinsulinaemic clamps are unethical and unfeasible to run at scale in a population of hyperglycaemic pregnant women. Giving a glucose load as part of an oral glucose load for the purposes of gestational diabetes diagnosis is valuable, but once the diagnosis is made, further glucose loads pose additional risk to the baby with no compensatory clinical benefit.

Although this is a limitation of our study, we used indices of insulin sensitivity that were used have been validated against clamps and intravenous methods in pregnancy [26, 27], and the index of insulin secretion validated against an intravenous method but not in pregnancy [28]. Our less labour-intensive indirect methods are more suited to relatively large, population-based studies like the present one, making our results relevant to study design for future observational and interventional studies.

### Significance of results upon pregnancy physiology and pathophysiology

Given that gestational diabetes is generally believed to result from reductions in both insulin sensitivity and secretion, and that GLP-1 concentrations were associated with both of these, an association with gestational diabetes may have been expected. However, a number of endocrine axes are altered in pregnancy, several of which can impact upon insulin sensitivity and secretion. Firstly, placental hormones and proteins such as human placental lactogen, human placental growth hormone and pregnancy-associated plasma protein A are thought to contribute to the regulation of insulin sensitivity in pregnancy [29]. Indeed prolactin and human placental lactogen may also increase glucose-stimulated insulin secretion [30]. Secondly there are certain maternal hormones such as oestradiol, progesterone, leptin, prolactin and cortisol that are present in increased concentrations in pregnancy which may also help regulate insulin sensitivity [29]. Adipokines, cytokines (e.g. tumour necrosis factor alpha) and exosomes may also regulate insulin sensitivity in pregnancy. This additional complexity may suggest that the overall relative contribution to the regulation of insulin sensitivity and secretion by incretins could be diminished in pregnancy. It may also explain the lack of association between GLP-1 concentrations and gestational diabetes in the present study.

Both fasting and OGTT 120 min GLP-1 concentrations were positively associated with fasting insulin concentrations and negatively with two indexes of insulin sensitivity (the Matsuda and Stumvoll indexes), in pregnant women with at least one recognised risk factor for gestational diabetes in the present study. This suggests that they were associated with insulin resistance. It is unlikely that raised GLP-1 concentrations caused insulin resistance, however, as outside of pregnancy they are thought more likely to be involved in increasing insulin sensitivity than reducing it [31]. A possible alternative explanation for the observed associations is that a confounding factor may both reduced insulin sensitivity and promoted GLP-1 secretion. One such factor could be dietary fatty acids which have been suggested to be able to both stimulate incretin secretion [32] and acutely reduce insulin sensitivity [33]. Whatever the actual mechanism, the associations were independent of significant associations with maternal BMIs, as adjusting for them did not alter significance.

Our results are consistent with the incretin effect of GLP-1 being preserved in pregnancy. As well as associations with indexes of insulin resistance, fasting and OGTT 120 min GLP-1 concentrations were both positively associated with the index of insulin secretion used (the ratio of areas under the insulin and glucose concentrations, 0–120 min). Although strongly associated with both insulin concentrations and indexes of insulin sensitivity and secretion, the GLP-1 concentrations were not associated with glucose concentrations at either of the time points in the OGTT. Assuming that there was an incretin effect of the GLP-1, it appears that the stimulation of insulin secretion was sufficient to overcome the insulin resistance in terms of the effects on the glucose concentrations. Although the associations with insulin but not glucose would suggest that in pregnancy GLP-1 may not have a major role in indirectly regulating glucose concentrations (via changes in insulin secretion), it may still have a role in indirectly regulating some of the non-glucose effects of insulin such as the stimulation of protein and fatty acid synthesis.

Our results provided limited evidence about the role of GIP in human pregnancy. Unlike the results for the GLP-1 concentrations, neither the fasting nor the OGTT 120 min GIP concentrations were associated with any of the key variables from the glucose-insulin axis. This could suggest the lack of, or at least a weakening of, an incretin effect for GIP in pregnancy. However, this was in a much smaller number of participants than for the GLP-1 associations, and the timing of sampling was suboptimal for assessing GIP’s function. Nonetheless, consistent with our findings, previous studies in mice have suggested a role for GLP-1 but not GIP in mediating the expansion of pancreatic beta cells and related adaptations to pregnancy [34].

### Significance of results with relevance to the existing literature

We identified no association between gestational diabetes and GLP-1 or GIP concentrations. This is at least partially consistent with some of the results from previous studies of incretins in gestational diabetes [9–14, 16] but not with all results from other published studies [9–13, 15]. The consistent results largely relate to the fasting concentrations of GLP-1 and GIP not being altered in women with gestational diabetes in the present study. The inconsistent results largely relate to there not being post-load alterations in GLP-1 and GIP concentrations in the present study. The differences in results between studies could relate to a number of different factors such as alterations in incretin concentrations being evident at different time points to those tested in the present study [10, 15], different glucose loads being used in the OGTTs [11, 15], and the use of a liquid meal test instead of a glucose load [13].

In summary, the present study suggests that the ability of GLP-1 to stimulate insulin production is preserved in pregnancy, but that this less important as a determinant of glycaemia. As well as having an incretin function, GLP-1 may therefore contribute to the regulation of insulin’s non-glucose functions such as fatty acid and protein biosynthesis in pregnancy.

## Data Availability

The datasets generated during and/or analysed during the current study are available from the corresponding author on reasonable request.
